# Undiagnosed interrupted aortic arch presenting with acute aortic dissection in a teenager: A rare case and successful repair

**DOI:** 10.1016/j.xjse.2025.100085

**Published:** 2025-10-18

**Authors:** İlke Keleş Selek, Çağla Canbay Sarılar, Birol Akdoğan, Sinan Bulut, Celal Caner Ercan, Sertaç Çiçek

**Affiliations:** a Department of Cardiovascular Surgery, Çorlu State Hospital, Tekirdağ, Turkey; b Department of Cardiovascular Surgery, Istanbul Faculty of Medicine, Istanbul University, Istanbul, Turkey; c Department of Radiology, Istanbul Faculty of Medicine, Istanbul University, Istanbul, Turkey

**Figure:**
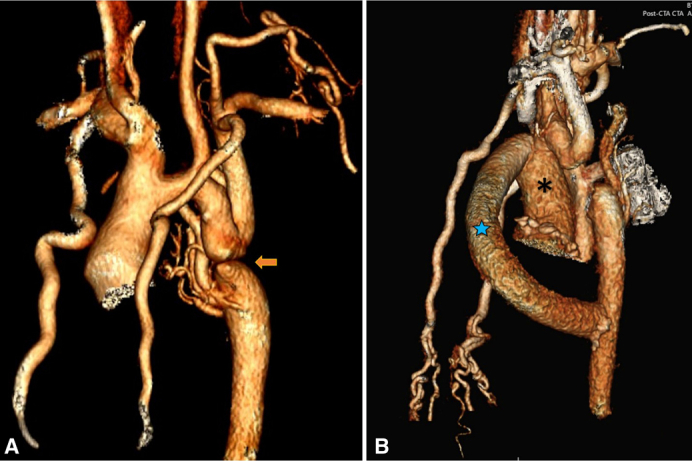
Three-dimensional volume-rendered computed tomography angiography (CTA) reconstructions.

**Central Message**

Extra-anatomic bypass offers a safe and effective solution for complex aortic pathologies when conventional reconstruction is not feasible.

Acute aortic dissection in the pediatric population is an extremely rare but life-threatening condition. The estimated incidence is less than 0.5 per million children annually.^1^ Most reported cases are associated with genetic aortopathies, including Marfan syndrome, Loeys-Dietz syndrome, and Ehlers-Danlos syndrome, although even in these cohorts, dissection during childhood or adolescence is uncommon. Interrupted aortic arch (IAA) is a rare congenital cardiac anomaly that represents approximately 1% of all congenital heart defects, with an incidence of about 3 per 1,000,000 live births.^2^ If left untreated, IAA is associated with 90% mortality by the first week of life, attributable to impaired systemic perfusion.

To our knowledge, no previously published report describes a patient who survived into adolescence without repair of a type A IAA and subsequently presented with an acute type A dissection. The current case provides an opportunity to highlight not only the exceptional nature of this clinical presentation but also the surgical strategy used for successful management—including extra-anatomic aortic reconstruction and preservation of the native aortic valve.

## Case Report

A 17-year-old man presented to the emergency department with a 20-hour history of chest and interscapular back pain. He was alert and hemodynamically stable but hypertensive, with systolic pressures above 180 mm Hg. There was no previous medical history of congenital heart disease, connective tissue disorder, or previous evaluation.

**Figure 1:**
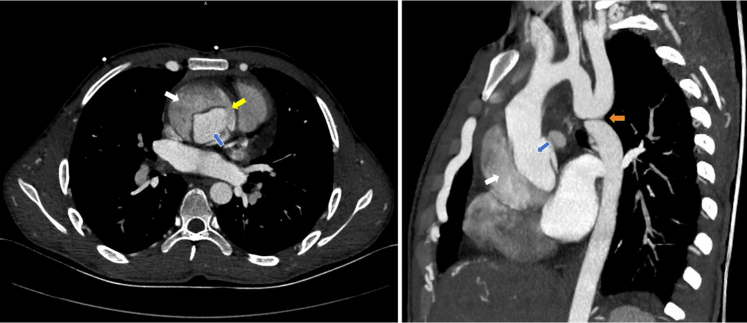
Preoperative computed tomography angiography demonstrating acute type A aortic dissection and interrupted aortic arch. Axial view: The *yellow arrow* shows the intimal tear in the ascending aorta; the *blue arrow* identifies the true lumen, and the *white arrow* indicates the false lumen. Sagittal view: The *blue* and *white arrows* correspond to the true and false lumens, respectively. The *orange arrow* shows the site of aortic interruption distal to the left subclavian artery.

Computed tomography angiography revealed a Stanford type A aortic dissection extending from the coronary sinuses to the brachiocephalic trunk. In addition, an interrupted aortic arch distal to the left subclavian artery (type A IAA) was identified (Figure 1). Perfusion of the descending aorta was maintained by well-developed collateral vessels, including the internal mammary and intercostal arteries.

The patient was urgently transferred to the operating room. Dual-site arterial cannulation was done: the femoral artery was used for lower body perfusion, whereas upper body perfusion was established via direct innominate artery cannulation. The patient was cooled to 24 °C to allow low-flow bypass during open distal anastomosis of the ascending aortic graft. This approach facilitated effective cerebral and systemic protection throughout the procedure. del Nido cardioplegia was used for myocardial protection.

Inspection of the ascending aorta revealed dissection involving all 3 aortic valve cusps, with an entry tear at the left coronary cusp. The dissection extended proximally to involve the aortic root, with clear separation of the intima and media down to the level of the coronary arteries. A formal valve-sparing root was not performed. Instead, we repaired the dissected aortic root while preserving the native aortic valve. The coronary arteries were not reimplanted, because they were unaffected. This combined approach—root repair-reinforcement and ascending aortic replacement—was chosen over a more extensive valve-sparing root replacement to reduce procedural complexity, especially in the emergency setting.

The affected root segment was reinforced using internal and external Teflon felt layers, which were bonded together with BioGlue to restore structural integrity and create a stable foundation. Commissural suspension sutures were then placed to reanchor the valve architecture. A 24-mm Dacron graft was subsequently anastomosed proximally to this reconstructed root and distally to the non-dissected portion of the ascending aorta. Because of a size mismatch between the graft and native aorta, the distal anastomosis was left partially incomplete on the left anterolateral aspect. To re-establish distal perfusion, a Gore-Tex graft was anastomosed end-to-side to the descending thoracic aorta via the posterior pericardium. This graft was tunneled through the left thorax and connected to the partially completed ascending aortic graft anastomosis, thereby completing the repair and restoring systemic flow. This extra-anatomic bypass avoided dissection through dense collateralized tissue and eliminated the need for total circulatory arrest.

**Figure 2:**
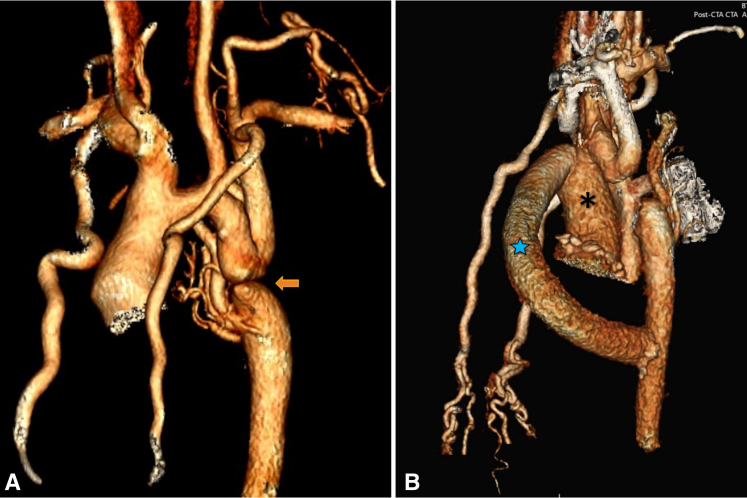
Three-dimensional volume-rendered computed tomography angiography reconstructions. A, The *orange arrow* indicates the interrupted aortic arch distal to the origin of the left subclavian artery. Prominent collateral vessels, including enlarged internal mammary arteries, are also visible. B, The *black asterisk* shows the supracoronary ascending aortic Dacron graft. The *blue star* shows the extra-anatomical Gore-Tex graft extending from the ascending aorta to the descending thoracic aorta distal to the interrupted segment.

The patient was weaned from bypass in stable condition and transferred to the intensive care unit. He was extubated at the fourth postoperative hour and was transferred to the floor second day. Postoperative imaging confirmed restoration of distal perfusion, minimal aortic valve regurgitation, and intact graft anastomoses (Figure 2). Recovery was uneventful, and the patient was discharged on the fifth postoperative day with antihypertensive medication.

## Discussion

This case highlights the convergence of 4 unique clinical dimensions: (1) the extreme rarity of aortic dissection in children and adolescents, even among those with genetic aortopathies; (2) the near-universal lethality of untreated IAA in infancy, with survival to age 17 years being almost extraordinarily rare, with only isolated cases reported in the literature; (3) the unprecedented combination of these 2 conditions; and (4) the role of individualized surgical strategies, particularly the use of an extra-anatomic bypass.

End-to-end anastomosis was not feasible in this case because of the long-segment interruption and the inelasticity of adult aortic tissue, which would have required extensive dissection and posed significant technical challenges in approximating the proximal and distal aortic segments. The choice of extra-anatomic bypass was guided by the anatomical realities of the case. An anatomic end-to-end graft reconstruction would have required a total arch replacement to bridge the gap between the ascending and descending aorta. In the context of acute dissection and extensive collateralization, this would have added prohibitive risk and technical complexity. The extra-anatomic bypass offered a safer and more expedient alternative, restoring distal perfusion without the need for circulatory arrest or arch intervention.^3^^,^^4^

IAA is typically diagnosed in neonates, and its presentation in adulthood is exceedingly uncommon, with fewer than 30 cases reported. Interestingly, although type B is more prevalent in infancy, most adult cases are classified as type A. This shift in anatomical pattern is believed to result from the gradual progression of undiagnosed aortic coarctation to complete luminal atresia, producing a functional interruption indistinguishable from congenital IAA. This hypothesis, supported by Sakkelaridis in his review of adult IAA cases, suggests that some late-presenting patients may have acquired interruption rather than a true congenital defect.^5^

The differentiation between congenital and acquired forms remains inherently challenging. Although this case meets the anatomical and physiological definition of a type A IAA, it is possible that the underlying lesion represents an acquired interruption arising from longstanding critical coarctation rather than a true congenital absence of aortic continuity. The absence of luminal continuity, extensive collateralization, and lack of previous intervention all support this diagnostic ambiguity.

The successful preservation of the native aortic root/valve further reduced operative complexity and eliminated the need for lifelong anticoagulation. Multidisciplinary preoperative planning and intraoperative adaptability were instrumental to the favorable outcome.

## Conclusions

This case illustrates an exceptionally rare intersection of congenital and acquired aortic pathology. Through tailored surgical planning, including extra-anatomic bypass and valve-sparing root repair, we achieved successful management of this life-threatening combination. The report underscores the adaptability of collateral circulation and the importance of individualized strategies in complex aortic reconstruction. Institutional review board approval was not required for this case report. Written informed consent was obtained from the patient for publication of this case report and any accompanying images.

## Conflict of Interest Statement

The authors reported no conflicts of interest.

The *Journal* policy requires editors and reviewers to disclose conflicts of interest and to decline handling or reviewing manuscripts for which they may have a conflict of interest. The editors and reviewers of this article have no conflicts of interest.
